# Epigenetics of hypertension as a risk factor for the development of coronary artery disease in type 2 diabetes mellitus

**DOI:** 10.3389/fendo.2024.1365738

**Published:** 2024-05-21

**Authors:** Raushan Zh Karabaeva, Tamara A. Vochshenkova, Nadiar M. Mussin, Rustam K. Albayev, Asset A. Kaliyev, Amin Tamadon

**Affiliations:** ^1^ Gerontology Center, Medical Center Hospital of the President’s Affairs Administration of the Republic of Kazakhstan, Astana, Kazakhstan; ^2^ General Surgery, West Kazakhstan Marat Ospanov Medical University, Aktobe, Kazakhstan; ^3^ Department for Natural Sciences, West Kazakhstan Marat Ospanov Medical University, Aktobe, Kazakhstan; ^4^ Department of Research and Development, PerciaVista R&D Co., Shiraz, Iran

**Keywords:** coronary artery disease, hypertension, type 2 diabetes mellitus, epigenetics, genetic variation, DNA methylation, histone, modification

## Abstract

Hypertension, a multifaceted cardiovascular disorder influenced by genetic, epigenetic, and environmental factors, poses a significant risk for the development of coronary artery disease (CAD) in individuals with type 2 diabetes mellitus (T2DM). Epigenetic alterations, particularly in histone modifications, DNA methylation, and microRNAs, play a pivotal role in unraveling the complex molecular underpinnings of blood pressure regulation. This review emphasizes the crucial interplay between epigenetic attributes and hypertension, shedding light on the prominence of DNA methylation, both globally and at the gene-specific level, in essential hypertension. Additionally, histone modifications, including acetylation and methylation, emerge as essential epigenetic markers linked to hypertension. Furthermore, microRNAs exert regulatory influence on blood pressure homeostasis, targeting key genes within the aldosterone and renin-angiotensin pathways. Understanding the intricate crosstalk between genetics and epigenetics in hypertension is particularly pertinent in the context of its interaction with T2DM, where hypertension serves as a notable risk factor for the development of CAD. These findings not only contribute to the comprehensive elucidation of essential hypertension but also offer promising avenues for innovative strategies in the prevention and treatment of cardiovascular complications, especially in the context of T2DM.

## 1 Introduction

Essential hypertension (EH) is a prevalent condition, affecting around 95% of adults diagnosed with high blood pressure. EH constitutes a substantial public health concern with considerable economic implications, accounting for an annual healthcare expenditure of approximately $50 billion in the United States alone (1–3). In 2013, EH was responsible for more than 350,000 deaths in the United States, making it a leading cause of mortality (2, 3). However, despite significant investments in healthcare, the precise etiology of EH remains elusive, impeding progress in treatment development. EH arises from a complex interplay of environmental factors, genetics, and epigenetics, which collectively influence biological pathways and contribute to the pathogenesis of hypertension. Notably, EH represents a major risk factor for renal injuries, cardiovascular pathologies, and cognitive dysfunction (4, 5).

While earlier perspectives diminished the role of genetics in hypertension, recent research has underscored the substantial impact of genetic and epigenetic determinants on blood pressure regulation and related conditions. Genome-wide association studies (GWAS) have identified common genetic variants associated with EH. However, owing to its polygenic nature, targeted, single-gene therapies for EH remain unavailable (6, 7).

Epigenetics, which explores the dynamic interplay between genetic factors and the environment, emerges as a critical player in the regulation of blood pressure. Epigenetic changes can be influenced by environmental stimuli such as nutrition, aging, and pharmaceuticals, and importantly, these changes possess the potential for reversibility (8, 9). This characteristic sets the stage for a spectrum of treatment possibilities distinct from those for genetic disorders. Epigenetics has garnered global attention, as evidenced by initiatives like the International Human Epigenome Consortium and the Human Epigenome Project (8, 10).

This comprehensive perspective aims to contribute to the understanding of the intricate interplay between genetics, epigenetics, and hypertension, with a specific focus on the chromosomal locus 9p21.3, in the context of CAD development in individuals with T2DM. The review focuses on the interaction between epigenetics and hypertension, specifically examining the role of DNA methylation, base methylation, gene methylation, and histone modification in hypertension pathogenesis. Additionally, the review discusses the therapeutic potential of miRNAs in hypertension and their role as diagnostic biomarkers. It also classifies epigenome modifications in hypertension based on pathophysiology and explores the epigenetic interplay between hypertension and CAD in T2DM. The review further investigates the environmental influences on epigenetics and hypertension, the relationship between pre-eclampsia, epigenetics, and hypertension, and provides insights into future directions in this field. This review focuses on discussing the role of microRNAs (miRNAs) and histone modifications in hypertension, excluding studies on long non-coding RNAs (LncRNAs).

## 2 Exploring the genetic significance of chromosomal locus 9p21.3 in hypertension, CAD, and T2DM

In this context, our manuscript proposes important additions, emphasizing the interaction of epigenetics and hypertension as a critical factor in the development of coronary artery disease (CAD) in type 2 diabetes mellitus (T2DM) (11). Specifically, we focus on the chromosomal locus 9p21.3, a genomic risk zone for cardiovascular diseases, which includes two distinct risk haplotypes for ischemic heart disease (IHD) and T2DM (12). These haplotypes, characterized by adjacent blocks of 50–100 single nucleotide polymorphisms (SNPs) separated by a recombination peak, exhibit linkage disequilibrium ensuring non-random joint inheritance for each disease (13). The potential overlap of T2DM SNPs in the CAD block makes the 9p21.3 locus a promising candidate for shared genetic risk for both CAD and T2DM (14). This condition sets the stage for the mechanical linkage of the 9p21.3 chromosomal locus to CAD and T2DM via ANRIL, the product of the cyclin-dependent kinase inhibitor gene (CDK2A/B) (15).

Moreover, the wide prevalence of risk haplotypes for hypertension, IHD, and T2DM (up to 50% of representatives of many populations) with a strong additive effect leads to at least 15% of cases of IHD and T2DM, making the chromosomal locus 9p21.3 the largest known genomic source of morbidity (16). The identification of a potential transcriptional regulatory mechanism in this locus, induced by the long non-coding mRNA ANRIL, suggests a common genetic signature for hypertension, CAD, and T2DM, alongside common environmental risks and clinical associations (17).

Additionally, the direct vascular and immunomodulatory functions of ANRIL, accelerating several signaling pathways (TNF-α-NF-kB-ANRIL and YY1-IL6/8), contribute to systemic inflammation, indirectly influencing the development of cardiometabolic diseases (18). This indicates a potential common genetic signature of hypertension, IHD, and T2DM at the level of the chromosomal locus 9p21.3 (16).

Furthermore, SNPs included in risk haplotypes for hypertension, coronary heart disease, and T2DM may be associated with differential expression of ANRIL splice variants (19). Determining their significance for the population at the level of significance for other populations could confirm the hypothesis of their association with differential expression of ANRIL splice variants for testing in subsequent studies (*in vivo* and *in vitro*) (20).

The locus 9p21.3, associated with risk haplotypes for hypertension, ischemic heart disease (IHD), and T2DM, represents a significant genetic source of morbidity, with potential implications for shared genetic risk factors and common environmental influences. Further research into the transcriptional regulatory mechanisms of this locus, particularly the role of the long non-coding mRNA ANRIL, may elucidate novel therapeutic targets and diagnostic biomarkers for cardiometabolic diseases.

## 3 Interaction between epigenetics and hypertension

**Figure 1:**
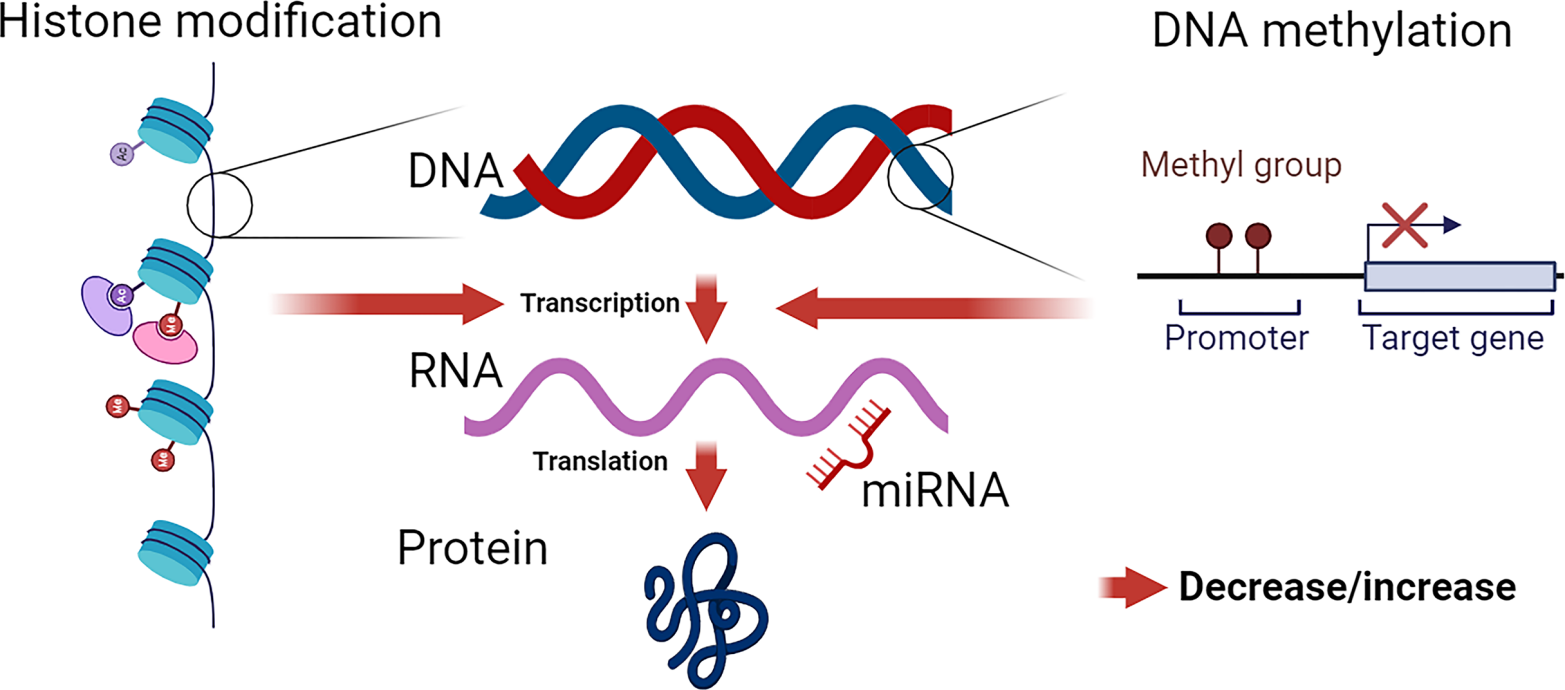
Epigenetic Modifications: Influence on Chromatin Structure and Gene Expression. DNA, wrapped around nucleosomes, comprises four pairs of histone proteins. Histones are susceptible to various epigenetic modifications, including acetylation, methylation, phosphorylation, sumoylation, and biotinylation. These modifications alter chromatin formation, leading to either an open (active) or closed (inactive) state, thereby modulating transcriptional activity. DNA methylation directly impacts DNA structure, influencing gene transcription. The effect of DNA methylation depends on the specific methylated site. miRNAs primarily target the 3’ UTR of mRNA (though 5’ targeting is possible), leading to the negative regulation of protein production by mRNA degradation or post-transcriptional regulation of mRNA stability.

Epigenetics refers to heritable changes in gene expression that occur without alterations in the DNA sequence. These changes are mediated by various mechanisms, including DNA methylation, histone modifications, and non-coding RNAs. Epigenetic modifications play a crucial role in regulating gene expression and have been implicated in the pathogenesis of various diseases, including hypertension (**Figure 1**).

DNA methylation is a well-established epigenetic mechanism in mammals (21), involving the covalent attachment of a methyl group to the 5’ position of a cytosine (C) within DNA (22). Genomic DNA frequently comprises short sequences of guanine (G) and cytosine dinucleotides linked by phosphodiester bonds, forming what are known as ‘CpG islands’ (23). Hypermethylation of CpG sites, typically occurring at cytosine bases, results in gene silencing. These epigenetic marks also play a pivotal role in determining active and inactive genomic regions by modulating the interplay between transcription factors and DNA (24). However, it is important to acknowledge that multiple cell types may exhibit similar levels of methylation, giving rise to diverse phenotypic expressions. Research has demonstrated a close association between the onset and severity of hypertension and DNA methylation levels, highlighting the imperative need for further exploration in this domain (23).

It is noteworthy that patho-clinical investigations related to EH and organ damage are often constrained by the limited availability of relevant animal samples. As a consequence, peripheral blood commonly serves as the preferred material for extensive human cohort studies. Nevertheless, the progression of the disease and its severity can influence the bio-metabolic processes governing DNA methylation, resulting in altered methylation patterns across different sample types (25). Some studies, such as the work by Kato et al., propose that blood and various tissues manifest analogous methylation patterns, suggesting that DNA methylation markers identified in blood mononuclear cells can serve as proxies for methylation profiles in other tissues (26). Conversely, investigations in rodents indicate that certain tissues may display distinct methylation patterns in response to specific pharmacological exposures. Hence, relying solely on the evaluation of blood mononuclear cells as epigenetic indicators may not consistently suffice for the assessment of diverse bodily samples (26).

### 3.1 Methylation of DNA

DNA methylation is a fundamental epigenetic mechanism that plays a crucial role in regulating gene expression. It involves the addition of a methyl group to the cytosine residue of a CpG dinucleotide, resulting in the formation of 5-methylcytosine. DNA methylation is essential for normal development and cellular differentiation, and aberrant DNA methylation patterns have been implicated in various diseases, including EH.

DNA methylation can be categorized into two primary types: gene-specific and global, depending on whether it pertains to the methylation status of a specific gene region or encompasses the overall level of 5-methylcytosine (5mC) across the entire genome (27). The methylation of DNA serves a dual purpose: it contributes to the preservation of genome integrity and exerts regulatory control over gene expression at the mRNA level (28). A multitude of studies have underscored the impact of DNA methylation on diverse pathophysiological processes, including EH, prompting extensive investigations into its role in hypertension and related cardiovascular disorders (29, 30). For a comprehensive overview of reported epigenetic modifications, such as DNA methylation, during the course of EH, please refer to **Table 1**.

**Table 1:** Review of epigenetic characteristics related to DNA methylation and hydroxyl-methylation in hypertension.

Epigenetic occurrence	Target gene	Severity	Study case	Effect	Reference
Methylation of DNA	5mC	High	Human placenta	Pre-eclampsia	(31)
DNA hydroxyl-methylation	5mC	High	Rat heart	Cardiac hypertrophy	(32)
Gene-specific DNA methylation					
HSD11B2	5mC	High	Human PBMCs	Renal sodium reabsorption	(33)
	5mC	Low	Rat adrenal gland	RAAS activation	(34)
NKCC1	5mC	Low	SHR hearts and aorta	Ionic transport	(35)
ACE	5mC	High	Human, PBMCs Rat liver and lungs *In vitro* HepG2, HT29, HMEC-1, SUT	RAAS activation	(36)
Atgr1a	5mC	Low	SHR endothelial cells	RAAS activation	(37)
PANX1	5mC	High	Heart and kidney in rat	Vitamin D3 deficiency	(38)

In EH, studies have shown alterations in both global DNA methylation levels and gene-specific DNA methylation patterns (39). Global DNA methylation refers to the overall level of methylation across the entire genome (40). Changes in global DNA methylation levels have been observed in hypertension, with some studies reporting global hypomethylation (41), while others have reported hypermethylation (42). These changes in global DNA methylation may contribute to the dysregulation of genes involved in blood pressure regulation and vascular function.

Gene-specific DNA methylation refers to the methylation status of specific genes or genomic regions (43). In EH, aberrant DNA methylation of genes related to the renin-angiotensin system (RAS), endothelial function, and vascular smooth muscle contraction has been reported (44). For example, hypermethylation of the angiotensinogen (AGT) gene promoter has been associated with increased blood pressure levels in EH patients (45).

### 3.2 Base methylation

**Figure 2:**
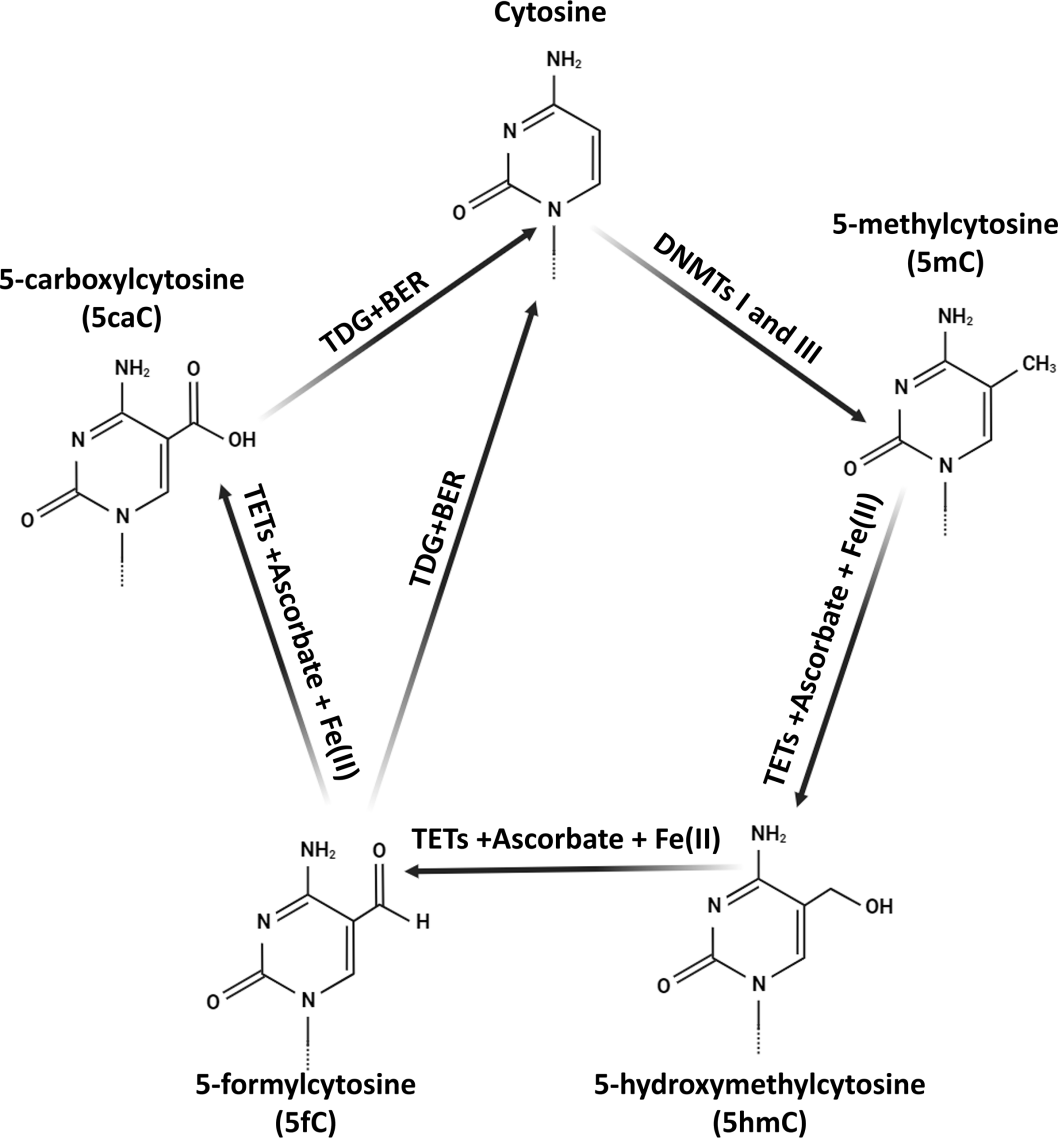
The biochemical pathways of 5-hydroxymethylcytosine (5hmC) in mammalian DNA. The synthesis of 5hmC is initiated by the TET protein-mediated oxidation (hydroxylation) of 5-methylcytosine (5mC). TET proteins along with co-factors Fe(II) and ascorbate generate 5hmC. Subsequent TET-driven oxidation of 5hmC consistently results in the formation of 5-formylcytosine (5fC) and 5-carboxylcytosine (5caC). These oxidized forms are then replaced with cytosine (C) through thymine-DNA glycosylase (TDG)-mediated base excision repair (BER). Additionally, DNA methyltransferases I and III (DNMTs I and III) facilitate the transfer of a methyl group to cytosine, producing 5mC.

About 3–4% of all cytosines in the genome, known as 5mC, are distributed throughout the DNA structure (**Figure 2**). Research efforts have revealed a connection between different pathological conditions and specific mRNA expression patterns, as well as gene-specific 5mC levels (46, 47). Furthermore, approximately 30 single nucleotide polymorphism (SNP) variants associated with hypertension have been identified via GWAS that are correlated with methylation markers, affirming the involvement of DNA methylation in EH (48). In parallel with 5mC, there exists a derivative known as 5-hydroxymethylcytosine (5hmC), which assumes a pivotal role in the demethylation process and is present in genomic DNA. Analogous to 5mC, 5hmC is pervasive throughout the mammalian genome, exhibiting distinct profiles across various tissues (49). A substantial body of research supports the role of 5hmC in gene regulation (50, 51). Additionally, a study in rats has provided initial evidence of a substantial correlation between hypertension and 5hmC levels (52). Nevertheless, current data linking EH to DNA hydroxymethylation remains limited, necessitating further research to elucidate the function of 5hmC in human EH.

The meticulous mapping of 5mC, which delineates DNA methylation patterns, offers a valuable blueprint for comprehending DNA functionality and stability. Previous investigations on DNA methylation have established a connection between 5mC levels and EH (53). Some studies have reported lower levels of 5mC in the DNA of whole blood in hypertensive patients, implying an inverse relationship between DNA methylation levels and the severity of EH (54). Conversely, pre-eclampsia, a form of hypertension occurring during pregnancy, has been positively associated with DNA hypermethylation (55). These findings strongly underscore the potential impact of DNA methylation on the development of EH. Studies in young males have indicated that DNA methylation may play a significant role in the development of EH, with its effects influenced by age (56). Furthermore, a recent animal study has demonstrated that cardiac hypertrophy, considered a relative index of EH following pressure overload, can be markedly ameliorated through the inhibition of DNA methylation, thereby emphasizing the role of DNA methylation in EH-associated cardiovascular damage in rat (32).

In summary, DNA methylation, particularly 5mC, is crucial in the development of EH. It is linked to various pathologies, mRNA expression patterns, and gene-specific 5mC levels. GWAS have associated hypertension-related SNP variants with methylation markers, supporting the role of DNA methylation in EH. Another important modification, 5hmC, is also implicated in EH and gene regulation, but further research is needed. Mapping 5mC patterns helps understand DNA functionality and stability, providing insights into potential EH treatments.

### 3.3 Gene methylation

Recent studies have primarily focused on investigating methylation patterns within specific gene regions, with gene-specific epigenetic modifications predominantly occurring within genomic regions called CpG islands, often situated in gene promoter regions (57). These CpG islands are found at the promoters of approximately 40% of genes, while other genomic regions also contain CpG sites Remarkably, in typical somatic cells, nearly 90% of CpG islands may undergo methylation, but those within promoter regions are relatively spared from such modifications (58, 59). Hypermethylation of CpG islands, particularly in promoter regions, plays a pivotal role in repressing gene transcription, as evidenced by numerous human and animal studies that consistently demonstrate an inverse relationship between 5mC levels and gene expression (60). It is important to note, however, that this correlation is not universally consistent and remains a subject of ongoing debate. Earlier research indicated a positive association between elevated levels of 5hmC and increased gene expression, suggesting that lower levels of 5mC result in gene silencing, while elevated levels of 5hmC are linked to gene activation (52, 61).

Furthermore, methylation of DNA at specific gene loci can influence the interplay between gene transcription factors and other epigenetic factors, such as histone modifications, leading to diverse expression patterns of the affected genes (62). Numerous genes documented in the literature have highlighted the role of epigenetic alterations in modulating biological and molecular processes relevant to EH. For example, the stimulation of sympathetic activity and the activation of the RAS, leading to altered sodium reabsorption in the kidney, are significant contributors to EH (63). Several RAAS genes are frequently studied in the context of EH. Additionally, the entire RAAS components can be found in the brain and may exhibit dysfunctional activity in various pathological conditions, including EH. In this context, the HSD11B2 gene, responsible for cortisol regulation, may experience suppression due to promoter hypermethylation, resulting in abnormal cortisol levels and the onset of EH (64). Studies in hypertensive rat models have further indicated a direct association between hypertension and hypermethylation of HSD11B2 (65). Moreover, in newborns, hypermethylation of the HSD11B2 gene, coupled with reduced HSD11B2 mRNA levels, suggests a potential mechanism for EH through abnormal renal sodium reabsorption (66–68).

Research has also demonstrated that DNA methylation in promoters can be modulated to enable the expression of genes such as Cytochrome P450 Family 11 Subfamily B Member 2 (CYP11B2), which plays a significant role in blood pressure regulation (69). In spontaneous hypertensive rats, hypomethylation in the gene promoter of the cotransporter, Na+/K+/2Cl (NKCC), leads to an increase in NKCC levels, correlated with postnatal hypertension. These findings collectively illustrate that dynamic changes in DNA methylation can influence gene expression, thereby impacting blood pressure regulation (70).

The RAAS pathway occupies a central role in EH, with genetic variants and altered epigenetic regulation of key genes in this pathway known to exert regulatory control over EH (71). Additionally, other genes associated with EH exhibit differential DNA methylation patterns associated with EH risk in a sex-, age-, and therapy-specific manner (**Table 2**).

**Table 2:** Epigenetic regulation of genes associated with essential hypertension (EH) in human (54, 72–75).

Gene	DNA methylation levels	Gene expression	EH risk
RAAS pathway genes	Altered	Upregulated	High
Estrogen receptor-α	Altered	Upregulated	High
PANX1	Altered	Upregulated	High
SULF1	Altered	Upregulated	High
NET	Altered	Upregulated	High
TIMP3	Altered	Upregulated	High
SERPINA3	Altered	Upregulated	High
CUL7	Altered	Upregulated	High
ADD1	Altered	Upregulated	High
Mfn2	Altered	Upregulated	High
IL-6	Altered	Upregulated	High
TLRs	Altered	Upregulated	High
IFN-γ	Altered	Upregulated	High
GCK	Altered	Upregulated	High

In summary, gene-specific DNA methylation and its impact on various biological and molecular pathways could play a pivotal role in EH. As a result, epigenetic markers hold the potential to estimate EH levels, associated risks, and enhance our understanding of its pathogenesis.

### 3.4 Histone modification

Nucleosomes are the basic units of chromatin, consisting of DNA wrapped around histone proteins. Histones are a family of proteins that play a crucial role in DNA packaging and gene regulation. Histone modifications, such as acetylation, methylation, phosphorylation, and ubiquitination, alter the structure of chromatin and regulate gene expression. These modifications can affect the accessibility of DNA to transcription factors and RNA polymerase, thereby influencing gene transcription.

Post-translational modifications occurring at the N-terminal histone tails of genomic DNA, such as acetylation, methylation, ubiquitination, and phosphorylation, represent integral epigenetic regulators associated with hypertension (23, 76). These histone modifications exert a profound influence on chromatin dynamics, often with discernible consequences. For example, histone acetylation primarily facilitates gene transcription, while histone deacetylation leads to gene silencing. However, such interactions are not universally reliable and can be context-dependent. Methylation of lysine at position 79 (H3K79) represses gene transcription, whereas methylation of histone arginine promotes it. Additionally, hypermethylation of lysine 9 (K9) results in gene suppression, whereas its hypomethylation allows gene transcription (77, 78). In these modifications, the interplay between epigenetic elements and histone tails controls DNA accessibility across the histones, thereby modulating the transcription of relevant genes. Moreover, these dynamics create an interactive environment for chromatin-modifying enzymes, enabling the specific regulation of gene expression (79).

Earlier studies have suggested that epigenetic elements can be passed down across generations (80). Recent research has even offered paternal epigenetic alterations in histones as potential indicators of offspring fertility, thereby proposing a suitable model for understanding how paternal epigenetic patterns can impact the health and development of offspring (81).

The latest investigations in hypertensive animal models, particularly rats, have indicated associations between histone modifications and the upregulation of ACE1 (82). A significant overexpression of ACE1 has also been reported in hypertensive offspring from lipopolysaccharide-treated rats, linked to histone H3 acetylation (H3Ac) within the ACE1 gene promoter region (83). Similarly, some studies have reported that hypertensive rats exhibited reduced levels of a specific gene (HSD11B2), attributed to the downregulation of H3K36 trimethylation, underscoring the role of histone modification in the regulation of chromatin structure in EH (83).

Studies conducted with human umbilical vein endothelial cells (HUVECs) have shown that hyperacetylation of H4K12 and H3K9, as well as di- and trimethylation of H3K4 at the promoter of the iNOS gene, contributes to an increase in blood pressure by modulating iNOS gene expression (84–86). These findings reveal that changes in iNOS mRNA gene expression due to histone acetylation can play a fundamental role in EH.

In this context, Cho et al. have demonstrated a correlation between hypomethylation and histone H3 in NKCC1 mRNA and protein levels following angiotensin II-triggered upregulation, indicating that epigenetic modifications can modulate the transcription of NKCC1 and related renal sodium reabsorption to influence blood pressure (87). Trouble-makers of telomeric silencing (TOT) have been linked to hypertension (88, 89). Specifically, TOT1a interaction with leukemia chromosome 9 (AF9) leads to H3K79 hypermethylation, suppressing the renal epithelial sodium channel (ENaC-α) and maintaining lower or normal blood pressure. However, aldosterone-mediated disruption of TOT1a-AF9 interaction results in H3K79 hypomethylation, leading to the activation of ENaC-α and ultimately, the development of severe hypertension (90).

Furthermore, Mehrotra et al. have reported that EH-mediated end-organ injuries, such as cardiac hypertrophy in rats subjected to a hypertensive condition and salt stimulation, result from higher levels of H3K4me3 and AcH4, alongside reduced levels of H3K9me3 and H3K27me3 in the overexpressed atrial natriuretic peptide (ANP) and brain natriuretic peptide (BNP) promoters (91).

Moreover, the central nervous system plays a crucial role in regulating histone modifications that affect arterial blood pressure. For example, the induction of melatonin neurons via H3 acetylation leads to increased hypertension in the medulla of the ventrolateral region through an upsurge in brainstem outflow, contributing to EH (92). Sympathetic nervous system-renal interactions direct hypertension in the presence of high salt levels. The activation of the sympathetic nervous system-renal axis can stimulate sodium retention, leading to both low and high levels of sodium reabsorption and increased systemic renin release, along with decreased kidney blood flow (93, 94). Additionally, the downregulation of the gene with-no-lysine kinase-4 (WNK4) is associated with salt-sensitive hypertension in rodents (95), and this downregulation is linked to histone modifications (96–98). Mu et al. have indicated that salt-induced acetylation of H3 and H4 results in WNK4 suppression, while concurrent overexpression of NCC leads to salt retention and subsequent hypertension (99). High-salt diets can also induce hypertension, which is related to lysine-specific demethylase-1 (LSD1) deficiency and the consequent hypermethylation of H3K9 (99).

Post-translational modifications occurring on DNA, particularly at the N-terminal histone tail sites, encompass processes such as ubiquitination, acetylation, methylation, and phosphorylation. These modifications play a pivotal role as epigenetic regulators associated with hypertension (88, 100). Each specific histone modification exerts a unique influence on chromatin structure, often yielding similar outcomes. For instance, histone acetylation predominantly facilitates gene transcription, while histone deacetylation tends to favor gene silencing. However, it is essential to acknowledge that this correlation is not universally consistent and remains a subject of ongoing debate. H3K79 acts as a repressor of gene transcription, whereas methylation of histone arginine can increase transcription. Furthermore, hypermethylation of histone K9 results in gene suppression, while its hypomethylation triggers gene transcription (82, 101). These modifications involve a tight interplay between epigenetic factors and histone tails, influencing the extent to which DNA is coiled around histones and, in turn, controlling the transcription of related genes. These dynamics also create sites of interaction for chromatin-altering enzymes, enabling the activation of gene expression (102). Additionally, past studies suggest the possibility of epigenetic markers being inherited across generations (103). A recent investigation even proposes a paternal mode of histone inheritance based on epigenetics, capable of impacting the fertility and health of future offspring (81).

In conclusion, histone acetylation and methylation can regulate chromatin and, subsequently, gene expression (**Table 3**). However, despite the evolving techniques and tools, the clinical application of histone epigenetics in prognostic and diagnostic approaches for EH remains challenging due to the complex nature of histone modifications.

**Table 3:** Summary of histone modifications and their role in essential hypertension (EH) (23, 76–78).

Histone modification	Role in EH
Histone acetylation	Promotes gene transcription by loosening chromatin structure
Histone methylation	Can activate or repress gene expression depending on the specific lysine residue and methylation state
Histone ubiquitination	Plays a role in DNA damage repair and gene expression regulation
Histone phosphorylation	Regulates chromatin condensation and gene expression

## 4 Role of miRNAs and epigenetics in hypertension pathogenesis: a brief overview

The miRNAs are small, non-coding RNA molecules that play a crucial role in post-transcriptional regulation of gene expression. They are typically about 21–23 nucleotides in length and function by binding to the 3’ untranslated region (UTR) of target messenger RNA (mRNA), leading to mRNA degradation or inhibition of translation. This process allows miRNAs to fine-tune the expression of target genes, impacting various cellular processes such as proliferation, differentiation, and apoptosis. In the context of EH, miRNAs have been implicated in the regulation of genes involved in blood pressure control and vascular function.

Transitioning to the realm of major blood pressure-regulating pathways, the RAAS stands as a well-established pathway, wherein angiotensin II governs fluid balance and blood pressure by stimulating aldosterone production. Research indicates that miR-21, a microRNA regulated by the RAAS-modulated AGT gene, may trigger aldosterone production in human adrenocortical cells under *in vitro* conditions, hinting at the potential role of miR-21 in human hypertension (104). Indeed, evidence points to a close connection between miR-21 and organ damage associated with hypertension (105). Moreover, miR-27a and miR-27b have been linked to the downregulation of ACE1 gene expression, while reduced levels of miR-330 can upregulate angiotensin II type-2 (AT2) receptor gene translation, thereby affecting the RAAS pathway in the fetal brain under malnourished conditions (106) (**Table 4**).

**Table 4:** Role of miRNAs in essential hypertension (EH): Target genes and effects.

miRNA	Effect on EH
miR-21	Stimulates aldosterone production, potentially contributing to hypertension (104)
miR-27a; miR-27b; miR-330	Downregulates ACE1 gene expression, affecting the RAAS pathway (106)
miR-5589; miR-539; miR-4436b-3p; miR-4500; miR-130b-5p; miR-4458; miR-4424; miR-497–3p; miR-4452; miR-374b-5p; miR-5584–3p	Associated with hypertension, potential diagnostic biomarker (107–110)

Experimental research conducted *in vitro* using HUVECs has illuminated that hyperacetylation of H4K12 and H3K9, alongside methylation (di- and tri-) of H3K4 within the eNOS gene promoter, triggers an increase in eNOS gene expression, which plays a pivotal role in blood pressure regulation (85, 111). This suggests that alterations in eNOS mRNA levels in response to histone acetylation may play a pivotal role in the context of EH.

In summary, the intricate web of genetic and metabolic signaling involved in EH implies the engagement of numerous miRNAs in the modulation of key genes. Consequently, based on our recent insights, miRNAs emerge as promising new biomarkers for unraveling the pathogenesis of EH, potentially leading to future clinical applications.

In a specific study, Cytoscape software was employed to construct 36 pairs of co-expression networks involving miRNAs and mRNAs, comprising 22 miRNAs and 25 mRNAs. Among these, 3 mRNAs (ARID3A, KIAA0513, and LRPAP1) exhibited connections with 3 distinct miRNAs, while 4 mRNAs (ADARB1, RASGRP1, ARF3, and FUCA2) showed associations with two miRNAs each. The remaining 18 mRNAs were linked to one miRNA each. Notably, this analysis revealed that LRPAP1, ARID3A, and KIAA0513 may have the potential to influence hypertension. A specific relationship was observed between has-miRNA-5589 and three target mRNAs. Additionally, miRNAs such as hsa-miR-539, hsa-miR4436b-3p, hsa-miR-4500, hsa-miR-130b-5p, hsa-miR-4458, hsa-miR-4424, hsa-miR-497–3p, hsa-miR-4452, hsa-miR374b-5p, and hsa-miR-5584–3p exhibited connections with 2 target mRNAs each, while the remainder showed connections with 1 target mRNA. This suggests that hsa-miRNA-5589–5p may play a particularly significant role in the onset of hypertension. In conclusion, these results indicate that mRNAs KIAA0513, LRPAP1, ARID3A, and hsa-miRNA-5589–5p can be considered as diagnostic biomarkers for patients with hypertension. Furthermore, the combination of hsa-miRNA-5589–5p and LRPAP1 may have diagnostic utility for hypertension. To explore the potential upstream regulation of these three key genes, the modulatory interaction between transcription factors and LRPAP1, ARID3A, and KIAA0513 was investigated (107–110). Among the 123 mRNAs associated with hypertension, only the CEBPA transcription factor gene was identified. Subsequent exploration of the public chromatin immunoprecipitation sequencing (ChIP-seq) Cistrome database (http://cistrome.org/db/#/) revealed the presence of CEBPA binding peaks upstream of both LRPAP1 and ARID3A. These findings further support the notion that CEBPA may play a role in regulating LRPAP1 and ARID3A in individuals with hypertension (112).

In summary, hypertension, a prevalent cause of cardiovascular disease worldwide, affects millions of people globally. Detecting hypertension in its early stages is crucial for effective blood pressure management. Moreover, miRNA and mRNA expression profiles were investigated for correlation between miRNA-mRNA networks and the development of hypertension. Firstly, Weighted Gene Co-expression Network Analysis (WGCNA) was employed to identify 123 mRNAs relevant to hypertension. Subsequently, Gene Ontology (GO) and Kyoto Encyclopedia of Genes and Genomes (KEGG) enrichment analyses were performed on these selected mRNAs. The results indicated that these mRNAs were enriched in terms related to interleukin 4 (IL4) regulation, signaling adaptor activity, and pathways such as tuberculosis, platelet activation, and the pentose phosphate pathway. Previous studies have linked tuberculosis infection to hypertension, suggesting a connection between these pathways and hypertension. Platelet activation is also related to hypertension, and the pentose phosphate pathway has relevance owing to its function in managing reactive oxygen species (ROS), which can contribute to oxidative stress-related hypertension. These findings support the involvement of the screened mRNAs in hypertension.

Subsequently, 35 Differentially Expressed miRNAs (DEMs) were identified between hypertension patients and healthy individuals, and 25 target mRNAs for these DEMs were identified, forming a miRNA-mRNA co-expression network. Among these mRNAs, ARID3A, LRPAP1, and KIAA0513, along with hsa-miRNA-5589–5p, demonstrated associations with hypertension. These could hold promise as suitable biomarkers for patients with hypertension. KIAA0513 has been linked to neuroplasticity, which can contribute to hypertension. ARID3A, a member of the ARID family, is applied as a bimolecular target for drugs in order to treat diseases, particularly hypertension. LRPAP1, involved in the suitable localization and folding of LDL receptor-related protein (LRP1), has known associations with hypertension and angiogenesis, a potential avenue for hypertension treatment. Additionally, public data revealed that CEBPA binding peaks were present upstream of LRPAP1 and ARID3A, strengthening the possibility of CEBPA’s regulatory role in hypertension (113). The miRNA-mRNA network constructed in our study may serve as a valuable diagnostic biomarker for hypertension patients. While miRNA-mRNA networks have previously been utilized to detect markers for various diseases, including hypertension, the regulatory networks involving LRPAP1, KIAA0513, and ARID3A have been less explored. Therefore, the findings of mentioned study provide further evidence of the potential diagnostic utility of these mRNAs and miRNAs in hypertension.

## 5 Therapeutic potential of miRNAs in hypertension

Currently, available therapies for pulmonary arterial hypertension (PAH) primarily focus on managing its symptoms rather than addressing its root causes, leading to less-than-optimal treatment outcomes and high mortality and morbidity rates associated with PAH. Thus, gaining a comprehensive understanding of the epigenetic mechanisms underlying PAH is imperative for the development of more effective treatments. Recent evidence has increasingly emphasized the significant role of miRNAs and long non-coding RNAs (lncRNAs) in the pathogenesis of PAH. Further research is essential to unlock the potential for innovative therapeutic approaches.

One study explored a treatment approach involving the administration of synthetic miR-204 to rats induced with PAH through monocrotaline (MCT). The intra-tracheal nebulization of synthetic miR-204 led to a reduction in pulmonary arterial blood pressure, decreased thickness of pulmonary arterial walls, and a decline in ventricular wall thickness. Importantly, this treatment resulted in a significant reduction in the activation of the STAT3–NFAT signaling pathway, leading to reduced proliferation and increased susceptibility to apoptosis in pulmonary arterial smooth muscle cells (PASMCs). Additionally, the downregulation of miR-204 in the buffy coat suggests its potential utility as a diagnostic marker for PAH (114).

Intriguing research investigated the impact of exosomes on animals (mice) with hypoxia-induced PAH and human pulmonary artery endothelial cells (hPAECs). Exosomes play a pivotal role in facilitating intercellular communication through paracrine signaling. In this study, researchers isolated exosomes from mesenchymal stromal cells (MSCs) derived from Wharton’s jelly in human umbilical cords and mouse bone marrow. These exosomes, termed MSC-derived exosomes (MSDEXs), were employed for treatment. MSDEX treatment increased the concentration of miR-204, a molecule typically decreased in PAH patient cells. Furthermore, this treatment inhibited the STAT3 signaling pathway, known to induce the miR-17/92 cluster while suppressing mRNA expression of miR-204 (115). This innovative therapeutic strategy enabled the modulation of various molecular pathways related to PAH, particularly those associated with miRNA dysfunction.

In another study, researchers transfected cells with miR-124 molecules, resulting in a reduction of lactic acid and glycolysis concentration, restoring them to normal levels. This transfection also normalized the rate of cell proliferation (116).

Chen et al. focused on modulating miR-29 levels in PASMCs obtained from transgenic mice with hereditary PAH (HPAH) resulting from a BMPR2 mutation (117). Over a two-week course, these mice received injections of anti-miR-29 (amiR-29). This therapeutic approach notably reduced pulmonary vascular resistance (PVR) and systolic pressure. It also reversed the enhanced muscularization observed in HPAH-affected mice. Furthermore, amiR-29 administration had beneficial effects on the molecular characteristics of the PASMCs, reducing insulin resistance and improving mitochondrial morphology, which is often compromised in HPAH (117).

Potus et al. restored normal angiogenesis in examined cells by increasing the concentration of miR-126 (118). In a 2015 study, they successfully corrected impaired angiogenesis in endothelial cells from skeletal muscles and increased microcirculation density through transfection with a miR-126 mimic. Additionally, in a 2015 study, scientists restored vascular density in cardiomyocytes obtained from PAH patients through miR-126 administration. Intravenous injection of the miR-126 mimic demonstrated advantages for rats with MCT-induced PAH, leading to improvements in ventricular performance and cardiac outcomes on echocardiography in the rodents after two weeks of such treatment (118).

Overall, these findings demonstrate the therapeutic potential of miRNAs and other epigenetic modifications in the treatment of hypertension. Further research is needed to fully elucidate the mechanisms involved and to develop effective therapeutic strategies for hypertension and related conditions.

## 6 miRNAs as diagnostic biomarkers in hypertension

Circulating miRNAs have garnered significant attention not only for their regulatory functions but also due to their accessibility and remarkable stability. As a result, these circulating miRNAs have emerged as promising diagnostic biomarkers for various pathological conditions, including cardiovascular diseases (119).

In a study conducted by Matshazi et al., an increased expression of miR-182–5p and miR-126–3p was notably observed in individuals with hypertension, whether screen-detected or previously diagnosed, in comparison to normotensive individuals. Nevertheless, significant differences in the mRNA levels of miR-30a-5p, miR-30e-3p, and miR-1299 were not detected between normotensive individuals and those with detected hypertension. Multivariable logistic regressions did not reveal an association between hypertension and the expression of miR-30e-3p and miR-1299. However, they did establish a connection between the expression of miR-126–3p, miR-182–5p, and miR-30a-5p with both screen-detected and previously known hypertension, especially in the latter group. This study, conducted within an African population, is significant as it represents the first instance of identifying differential expression of miRNAs in whole blood based on blood pressure status. These miRNAs may serve as a potential panel of diagnostic biomarkers for hypertension. Moreover, the research reaffirmed prior findings concerning miR-126 and the miR-30 family, highlighting their potential involvement in hypertension development. Further exploration of these non-coding RNAs may open new avenues for prognosis and therapy in the context of cardiovascular diseases (120).

In another study, Yang et al. evaluated miR-505 as a prospective diagnostic biomarker for hypertension. Their findings provided evidence of miR-505’s prognostic relevance in hypertension-related inflammation. Clinical data indicated a positive correlation between plasma levels of miR-505, systolic blood pressure, and CRP. CRP serves as an inflammatory marker linked to target organ damage in hypertensive patients, such as vascular alterations and cardiovascular events. The positive association of plasma miR-505 with CRP aligns with clinical outcomes suggesting miR-505’s pro-inflammatory role, further substantiating its link to systemic inflammation in hypertension (119).

Furthermore, a study led by Charkiewicz and colleagues examined 88 men with hypertension, assessing various miRNAs in their serum levels. Elevated levels of miR-145–5p, miR-1–3p, and miR-423–5p pointed to the potential involvement of these specific miRNAs in hypertension. It is important to note that limited studies have explored this area, making comparisons with existing literature somewhat challenging. MiR-145–5p and miR-1–3p are believed to safeguard vascular smooth muscle cells by regulating processes related to proliferation and migration. Interestingly, circulating miR-423–5p levels were found to be reduced shortly after a severe myocardial infarction, followed by an increase after five months in the same group of patients, highlighting the dynamic nature of miRNA levels. Discrepancies in miRNA levels among various research centers may stem from differences in study methods, patient selection, age, sex, or ethnicity (121). Liang et al. assessed 1,141 miRNAs in two subgroups of genotype-positive hypertension patients and identified 20 miRNAs with potential significance in patients with hypertension (122).

## 7 Epigenome modifications in hypertension: a classification based on pathophysiology

### 7.1 Salt-sensitive and salt-resistant hypertension

Salt-sensitive hypertension is characterized by an exaggerated blood pressure response to changes in salt intake, while salt-resistant hypertension is less affected by salt intake (123). Recent studies have suggested that epigenetic modifications, particularly DNA methylation, may play a role in the development of salt-sensitive hypertension by regulating genes involved in sodium handling and blood pressure regulation (23). Understanding the epigenetic basis of salt-sensitive and salt-resistant hypertension could lead to the development of targeted therapies that modulate these epigenetic mechanisms to treat or prevent hypertension based on individual salt sensitivity (124).

### 7.2 RAS-dependent hypertension

The RAS plays a crucial role in blood pressure regulation, and epigenetic modifications have been implicated in its dysregulation in hypertension (125). DNA methylation and histone modifications have been shown to regulate the expression of genes in the RAS, such as AGT and ACE, influencing blood pressure control (126). Targeting epigenetic modifications in the RAS could be a promising approach for the treatment of RAS-dependent hypertension (127). Drugs that modulate DNA methylation or histone acetylation patterns could potentially restore RAS balance and reduce blood pressure (128).

### 7.3 Vascular function-dependent hypertension

Epigenetic modifications have also been implicated in the regulation of vascular function in hypertension (82). Changes in DNA methylation and histone modifications can alter the expression of genes involved in vascular smooth muscle contraction, endothelial function, and vascular remodeling, contributing to hypertension development (129). Future research should focus on elucidating the specific epigenetic changes associated with vascular function-dependent hypertension and developing targeted therapies to restore vascular function and reduce blood pressure.

## 8 Epigenetic interplay between hypertension and coronary artery disease in T2DM

### 8.1 Epigenetic links between hypertension and CAD

#### 8.1.1 Shared pathways

Recent studies have highlighted the role of epigenetic modifications, such as DNA methylation and histone modifications, in the regulation of endothelial function in both hypertension and CAD (130). Aberrant DNA methylation patterns in genes related to endothelial function, such as eNOS and ET-1, have been associated with endothelial dysfunction, a common feature of both conditions (131). Epigenetic changes, particularly histone modifications and non-coding RNAs, have been implicated in the regulation of inflammatory pathways in hypertension and CAD (130). For example, miRNAs have been shown to modulate the expression of inflammatory genes, such as IL-6 and TNF-alpha, contributing to inflammation in both conditions (132). Epigenetic modifications, including DNA methylation and histone acetylation, have been linked to oxidative stress, a key mechanism underlying endothelial dysfunction and vascular damage in hypertension and CAD (133). These epigenetic changes can alter the expression of genes involved in antioxidant defense mechanisms, exacerbating oxidative stress in both conditions (134).

#### 8.1.2 Epigenetic biomarkers

Recent studies have identified specific DNA methylation changes associated with both hypertension and CAD, suggesting that DNA methylation patterns may serve as potential biomarkers for cardiovascular risk assessment in individuals with T2DM (135). For example, hypermethylation of the ACE gene has been linked to increased risk of hypertension and CAD (136). Histone modifications, such as acetylation and methylation, have also been proposed as potential biomarkers for cardiovascular risk in individuals with T2DM (137). Altered histone acetylation patterns in genes related to inflammation and oxidative stress have been associated with increased risk of hypertension and CAD (138).

### 8.2 Epigenetic factors in the development of CAD in T2DM

#### 8.2.1 Synergistic effects

Recent evidence suggests that the combination of hypertension, epigenetic modifications, and T2DM can lead to a greater risk of developing CAD than each factor alone (139). The synergistic effects of these factors may involve complex interactions between genetic and environmental factors, leading to dysregulation of pathways related to endothelial function, inflammation, and oxidative stress (140). Epigenetic modifications associated with hypertension and T2DM, such as DNA methylation and histone modifications, may interact synergistically to alter gene expression patterns related to CAD development (141). For example, hypermethylation of genes involved in lipid metabolism and endothelial function may exacerbate atherosclerosis in individuals with both conditions (142).

#### 8.2.2 Clinical implications

Recognizing the synergistic effects of hypertension, epigenetic modifications, and T2DM on CAD development is crucial for effective risk assessment and management strategies (143). Clinicians should consider these factors when evaluating cardiovascular risk in patients with T2DM. Understanding the epigenetic basis of hypertension as a risk factor for CAD in T2DM opens up new avenues for therapeutic interventions. Targeting specific epigenetic pathways implicated in CAD pathogenesis may offer novel therapeutic strategies to reduce cardiovascular risk in this population. For example, drugs that modulate DNA methylation or histone acetylation patterns could potentially be used to prevent or treat CAD in individuals with T2DM and hypertension (144).

### 8.3 Future directions

Future studies should aim to elucidate the specific epigenetic changes that contribute to the development of CAD in individuals with hypertension and T2DM. By identifying these changes, researchers can gain insights into the underlying mechanisms of CAD in this population and identify potential therapeutic targets. Additionally, these studies should focus on identifying epigenetic biomarkers that can be used for risk stratification in individuals with hypertension and T2DM. These biomarkers could help clinicians identify patients at higher risk of developing CAD and tailor treatment strategies accordingly.

Understanding the epigenetic basis of hypertension and CAD in individuals with T2DM may pave the way for precision medicine approaches. These approaches could target specific epigenetic pathways to reduce cardiovascular risk in this population. For example, drugs that modulate DNA methylation or histone modifications could be developed to target these pathways. Future studies should focus on validating epigenetic biomarkers that have been identified as potential targets for precision medicine approaches. Validation studies will be crucial in determining the effectiveness of these biomarkers in predicting CAD risk and guiding treatment decisions.

The concept of personalized medicine, which considers individual genetic and epigenetic profiles, holds promise for the development of targeted therapies for CAD in patients with T2DM and hypertension. Future research should focus on identifying specific epigenetic signatures associated with CAD in this population and developing personalized treatment approaches based on these signatures. However, the implementation of personalized medicine approaches in clinical practice may present challenges, including the need for robust validation studies, development of cost-effective testing methods, and integration of genetic and epigenetic data into clinical decision-making.

## 9 Environmental influences on epigenetics and hypertension

### 9.1 Diet influences on epigenetics and hypertension

Diets rich in nutrients such as folate, vitamin B12, and other methyl donors can influence DNA methylation patterns, potentially affecting genes involved in blood pressure regulation (145). Conversely, diets high in salt or fat may lead to epigenetic changes that contribute to hypertension (146). Studies have shown that dietary patterns, such as the Dietary Approaches to Stop Hypertension (DASH) diet, which is rich in fruits, vegetables, and low-fat dairy products, can lead to changes in DNA methylation associated with lower blood pressure (147).

### 9.2 Pollution influences on epigenetics and hypertension

Exposure to environmental pollutants, such as particulate matter, polycyclic aromatic hydrocarbons (PAHs), and heavy metals, can alter DNA methylation patterns and gene expression, potentially contributing to the development of hypertension (148). Epidemiological studies have linked exposure to air pollution with changes in DNA methylation of genes related to inflammation and oxidative stress, which are implicated in hypertension (149).

### 9.3 Ethnicity and geography influences on epigenetics and hypertension

Ethnicity and geographic location can impact epigenetic patterns through differences in lifestyle, diet, cultural practices, and environmental exposures, all of which may contribute to variations in hypertension prevalence among different populations (23). Studies have shown ethnic differences in DNA methylation patterns associated with hypertension-related genes, suggesting that genetic and environmental factors unique to certain populations may play a role in hypertension disparities (150).

## 10 Relationship between pre-eclampsia, epigenetics, and hypertension

Pre-eclampsia is a hypertensive disorder that occurs during pregnancy and is characterized by high blood pressure and proteinuria (151). It shares some pathophysiological features with EH, suggesting a potential common etiology involving genetic and epigenetic factors (152). Epigenetic modifications, such as DNA methylation, histone modifications, and non-coding RNA expression, play a crucial role in the pathogenesis of pre-eclampsia (153). These epigenetic changes can alter the expression of genes involved in vascular function, inflammation, and placental development, contributing to the development of hypertension in pre-eclamptic women (154). Current research in this field has focused on elucidating the specific epigenetic changes associated with pre-eclampsia and their role in the pathogenesis of the disorder (155). Studies have identified differential DNA methylation patterns in placental tissue and maternal blood of women with pre-eclampsia compared to healthy pregnant women, suggesting that these epigenetic changes may contribute to the development of the disorder (156). Recent research has also provided insights into the pathogenesis of pre-eclampsia, highlighting the role of aberrant placentation, oxidative stress, and immune dysregulation (157). These processes are influenced by epigenetic mechanisms, further implicating epigenetic factors in the development of hypertension in pre-eclampsia. Further research is needed to unravel the complex interplay between genetic and epigenetic factors in pre-eclampsia and hypertension. Longitudinal studies that follow women with pre-eclampsia beyond pregnancy may provide valuable insights into the long-term effects of epigenetic changes on hypertension risk later in life.

## 11 Conclusion

The amalgamation of evidence gleaned from diverse databases provides crucial insights into EH, a multifaceted condition intricately regulated by multiple genes. These genes undergo intricate control through diverse epigenetic mechanisms, including histone modifications, DNA methylation, and miRNAs. A noteworthy proportion of EH-associated genes harbor CpG sites susceptible to DNA methylation and are subject to the regulatory influence of various miRNAs, thereby being responsive to a spectrum of epigenetic determinants. The synergistic interplay of these epigenetic modifications holds immense potential for advancing the diagnostic and therapeutic paradigms of EH.

Advanced technologies such as Genome-Wide Association Studies (GWAS) and Epigenome-Wide Association Studies (EWAS) empower the exploration of epigenetic and genetic variations across different forms of hypertension. Through these methodologies, novel loci influencing blood pressure regulation have been unveiled, with ongoing research poised to uncover further insights. The expansive realm of epigenetics continuously uncovers additional hereditary variations intertwined with hypertension.

In a concerted effort to unravel the intricate crossroads of genetic and epigenetic regulatory elements, researchers aspire to deepen their comprehension of hypertension’s pathogenesis, paving the way for personalized therapeutic interventions. High-throughput techniques, including whole-genome and exome sequencing, offer a holistic perspective for the simultaneous exploration of multiple risk variants.

Understanding the role of environmental influences on epigenetics is essential for elucidating the mechanisms underlying hypertension. By considering the impact of diet, pollution, and ethnicity on epigenetic modifications, we can gain a more comprehensive understanding of the multifactorial nature of hypertension and develop more targeted strategies for its prevention and management.

In conclusion, a comprehensive understanding of hypertension’s risk factors, incorporating both epigenetic and genetic markers, forms an imperative foundation for tailoring personalized therapeutic strategies. This integrated approach holds promise for advancing precision medicine in the management of EH and related cardiovascular complications.

## Author contributions

RZ: Conceptualization, Funding acquisition, Project administration, Writing – review & editing. TV: Conceptualization, Data curation, Formal Analysis, Writing – review & editing. NM: Data curation, Methodology, Resources, Writing – review & editing. RA: Methodology, Validation, Writing – review & editing. AK: Data curation, Methodology, Resources, Writing – review & editing. AT: Conceptualization, Formal Analysis, Investigation, Methodology, Resources, Writing – original draft.
